# Supplementary material for: Descriptive Epidemiology of Melanoma Diagnosed between 2010 and 2014 in a Colombian Cancer Registry and a Call for Improving Available Data on Melanoma in Latin America
Source: Cancers (Basel). 2023 Dec 15;15(24):5848. doi: 10.3390/cancers15245848 (PMC10741499; doi:10.3390/cancers15245848)
Supplement: Supplementary file 1 [file cancers-15-05848-s001.zip › cancers-2734337-supplementary.pdf]

Supplementary table S1: Distribution of cutaneous melanomas by anatomical localization and sex in AMB PBCR, 2010-2014

**Anatomical localization**	**Male**	**Female**	**Total**
Head and neck	9 (16.4%)	8 (13.8%)	17 (15.0%)
Trunk	17 (30.9%)	7 (12.1%)	24 (21.2%)
Upper limbs	10 (18.2%)	5 (8.6%)	15 (13.3%)
Lower limbs	14 (25.4%)	34 (58.6%)	48 (42.5%)
Unknown	5 (9.1%)	4 (6.9%)	9 (8.0%)
**Total**	**55 (100.0%)**	**58 (100.0%)**	**113 (100.0%)**
